# NHANES‐Derived Machine Learning Model for Early Identification of Frailty Risk in CKM: Optimizing Resource Allocation Through Predictive Analytics

**DOI:** 10.1155/cdr/7296443

**Published:** 2026-03-18

**Authors:** Wenlong Ding, Caoyang Fang, Fachao Shi, Lei Fang, Qin Cui, Zheng Wang

**Affiliations:** ^1^ Department of Cardiology, Xuancheng Hospital Affiliated to Wannan Medical College (Xuancheng People′s Hospital), Xuancheng, Anhui, China; ^2^ Department of Emergency, The First Affiliated Hospital of USTC, Division of Life Sciences and Medicine, University of Science and Technology of China, Hefei, Anhui, China, ustc.edu.cn; ^3^ Department of Cardiology, Maanshan People′s Hospital, Maanshan Hospital Affiliated to Wannan Medical College, Maanshan, Anhui, China; ^4^ Department of Geriatrics Center, Tongling People′s Hospital, Tongling, Anhui, China, eurofinsa.com; ^5^ Department of Cardiology, The Second People′s Hospital of Hefei, Hefei Hospital Affiliated to Anhui Medical University, Hefei, Anhui, China, ahmu.edu.cn

**Keywords:** cardiovascular–kidney–metabolic, CKM, frailty, machine learning, NHANES, predictive model

## Abstract

**Background:**

CKM encompasses a complex group of disease states involving cardiovascular, kidney, and metabolic dysfunction. Frailty is a significant health issue among older adults and is closely associated with adverse outcomes. However, there is currently a lack of predictive tools specifically designed for assessing frailty risk in middle‐aged and older patients with CKM Stages 0–3.

**Objective:**

This study is aimed at developing a machine learning model to predict frailty risk in this population and to identify key predictive factors.

**Methods:**

Using data from NHANES 2007–2018, a total of 6749 participants aged 40 years and older were included. Feature selection was conducted using LASSO regression, and the cohort was randomly divided into training and testing sets in a ratio of 7:3. Five machine learning models LR, LightGBM, XGBoost, RF, and DT were compared. Model performance was evaluated via 10‐fold cross‐validation, and the optimal model was selected based on accuracy, AUC, precision, recall, and F1 score. Feature importance was further explored using SHAP values.

**Results:**

Among the 6749 participants, 1427 (21.14%) were identified as frail. LASSO regression identified 21 important variables, which were used to construct the five machine learning models. The RF model achieved the best performance in predicting frailty risk, as demonstrated by its AUC (0.90), accuracy (0.81), precision (0.54), recall (0.84), and F1 score (0.66). Decision curve analysis and calibration plots further confirmed the clinical utility of this approach. SHAP analysis revealed that the most significant predictors were PIR, antihypertensive medication use, hemoglobin, red blood cell count, albumin, and diabetes.

**Conclusion:**

The machine learning model developed in this study can effectively predict the risk of frailty in middle‐aged and older adults with CKM Stages 0–3, providing a valuable tool for early identification of high‐risk individuals in clinical practice. This model may help optimize healthcare resource allocation, guide‐targeted interventions, and improve health management for patients with CKM.

## 1. Introduction

Cardiovascular–kidney–metabolic (CKM) syndrome is a complex disease state involving interactions among multiple organ systems, including cardiovascular disease, chronic kidney disease, and metabolic disorders such as diabetes and obesity [1]. Recent studies have shown that these three systems not only share common pathophysiological mechanisms such as low‐grade chronic inflammation, oxidative stress, and endothelial dysfunction but are also interconnected in ways that progression in one system can accelerate deterioration in others [2]. CKM syndrome can be staged according to the number of affected systems, offering a new perspective for assessing disease burden and risk stratification [3].

Frailty is a central concept in geriatric medicine, defined as a state of decreased physiological reserve and diminished resilience to stressors [4]. The frailty phenotype proposed by Fried et al., consisting of unintentional weight loss, self‐reported exhaustion, reduced physical activity, slow walking speed, and weakened grip strength, is one of the most widely used frailty assessment criteria [5]. A large body of research has demonstrated that frailty is associated with a range of adverse health outcomes, including increased risk of falls, hospitalization, functional dependence, nursing home admission, and mortality, imposing a substantial burden on patients, families, and the healthcare system [6, 7].

Evidence suggests that each component of the CKM syndrome, even when occurring alone, is linked to a higher risk of frailty. Cardiovascular disease may contribute to muscle dysfunction and frailty by reducing cardiac output, decreasing skeletal muscle perfusion, and promoting inflammation [8]. Chronic kidney disease leads to protein‐energy wasting and muscle atrophy through mechanisms such as accumulation of uremic toxins, metabolic acidosis, vitamin D deficiency, and secondary hyperparathyroidism [9]. Metabolic disorders, especially diabetes, accelerate the loss of muscle mass and function via insulin resistance, vascular complications, and neuropathy [10]. However, systematic research on the association between different CKM stages classified by the number of involved systems and frailty remains limited, particularly regarding the lack of reliable predictive tools for identifying high‐risk individuals within this complex population [11].

Traditional risk assessment methods, such as linear or logistic regression models, have limitations in handling complex, multidimensional, and nonlinear relationships [12]. With advances in computing power and algorithm development, machine learning techniques have shown great potential in medicine, as they can process large‐scale datasets and identify intricate patterns and interactions that conventional statistical methods might overlook [13]. Machine learning has already demonstrated superior performance compared with traditional approaches in predicting the risk of cardiovascular disease [14], kidney disease [15], and diabetes [16].

Previous NHANES‐based studies have successfully applied machine learning methods to predict frailty risk in general populations, demonstrating the potential of these approaches for population health management [17, 18]. However, these studies focused on heterogeneous populations without considering the specific pathophysiological context and risk factor patterns unique to CKM syndrome. The present study addresses several critical gaps in the existing literature:

First, this study specifically targets CKM Stages 0–3 patients, a well‐defined population with distinct pathophysiological characteristics that differ from the general population. The CKM framework recognizes the interconnected nature of cardiovascular, kidney, and metabolic dysfunction, requiring specialized predictive approaches that account for multisystem interactions rather than treating each condition in isolation.

Second, although previous studies have developed predictive models, they often lack the clinical interpretability necessary for practical implementation. Our study employs SHAP (Shapley Additive exPlanations) analysis to provide transparent, interpretable machine learning predictions that clinicians can understand and trust. This interpretability is crucial for clinical adoption, as it allows healthcare providers to understand not just the prediction outcome but also the underlying factors driving the risk assessment.

Third, the staged approach of CKM syndrome (Stages 0–3) provides a unique opportunity for risk stratification and targeted interventions at different disease progression points. By excluding CKM Stage 4 patients (those with established cardiovascular disease), our model focuses on the earlier stages where preventive interventions may be most effective, filling a critical gap in early identification and prevention strategies.

Fourth, our study integrates comprehensive multidimensional data including socioeconomic factors, clinical parameters, laboratory biomarkers, and medication use patterns specifically relevant to the CKM population. This holistic approach captures the complex interplay of factors that influence frailty risk in this specific patient population, providing more precise and actionable predictions than generic population‐based models.

However, studies applying machine learning to predict frailty risk specifically in the context of CKM syndrome are still scarce. The NHANES is an ongoing, large‐scale, nationally representative survey that collects comprehensive health and nutritional information from the US population. Its dataset includes detailed demographic, clinical, laboratory, and functional assessment variables, making it an ideal platform to explore the relationship between CKM syndrome and frailty. Leveraging the rich data resources of NHANES, together with advanced machine learning methods and interpretable artificial intelligence techniques, offers the potential to develop precise, clinically applicable frailty risk prediction models specifically designed for the CKM population.

The primary aim of this study is to develop and validate an interpretable machine learning‐based predictive model for frailty risk specifically among middle‐aged and older adults with CKM Stages 0–3, using NHANES data from 2007 to 2018. This model is intended to facilitate early identification of high‐risk individuals within this specific population, guide‐targeted interventions, and ultimately improve health outcomes through personalized risk stratification and management strategies for patients with CKM syndrome.

## 2. Methods

### 2.1. Study Design and Data Sources

This study utilized a cross‐sectional design based on data from the NHANES collected between 2007 and 2018. NHANES is a nationwide, ongoing survey initiated by the National Center for Health Statistics of the Centers for Disease Control and Prevention, aimed at assessing the health and nutritional status of the US population. The survey employs a complex, multistage probability sampling design to ensure that the sample is representative of the civilian, noninstitutionalized population in the United States. Data are collected through household interviews, laboratory tests, and physical examinations. For this study, data from six consecutive NHANES cycles (2007–2008, 2009–2010, 2011–2012, 2013–2014, 2015–2016, and 2017–2018) were combined for analysis. NHANES protocols have been approved by the CDC Institutional Review Board, and all participants provided informed consent. Only publicly available, deidentified data were used in this study; therefore, additional ethical approval was not required.

### 2.2. Study Population

**Figure 1:**
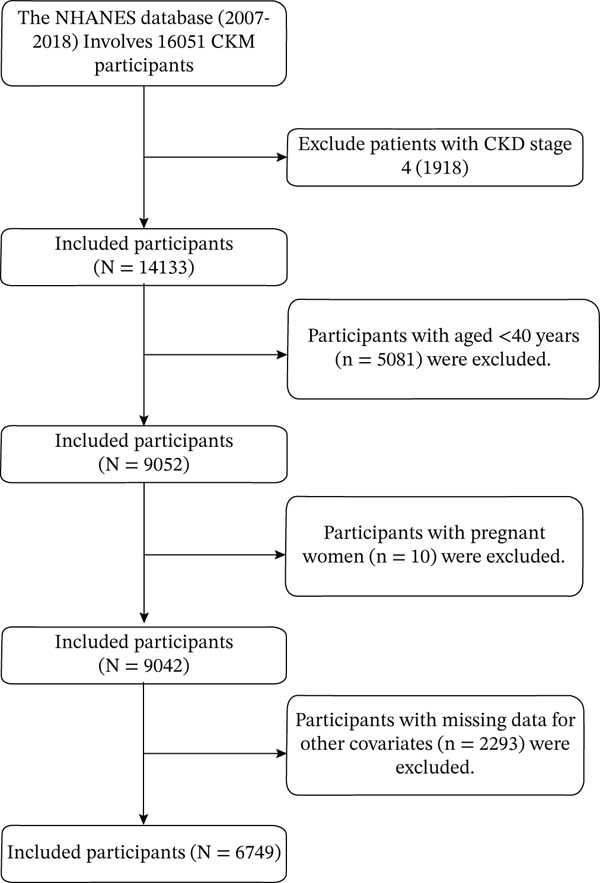
Study flow chart.

This study included adult participants aged 40 years and older from the NHANES 2007–2018 data. Exclusion criteria were as follows: (1) CKM Stage 4 participants were excluded (*n* = 1918); (2) participants aged < 40 years were excluded (*n* = 5081); (3) pregnant women were excluded (*n* = 10); (4) cases with missing data for other covariates (*n* = 2293). A total of 6749 participants met the inclusion criteria (Figure 1). Missing data patterns were systematically analyzed to assess potential selection bias. Variables with > 20% missing data in any demographic or clinical subgroup were flagged for sensitivity analysis. Participants were excluded from the primary analysis only if they had missing data for key variables essential to CKM staging or frailty assessment. A comprehensive comparison of baseline characteristics between included and excluded participants was performed to evaluate the representativeness of the final analytical sample and to identify potential limitations in generalizability.

### 2.3. Definition of CKM Syndrome

CKM staging was defined according to the latest consensus, based on medical history, laboratory tests, and physical examination data collected in NHANES [19, 20]. CKM Stage 0: Individuals who are healthy without underlying diseases or risk factors. CKM Stages 1–3: Individuals who are defined by the presence of metabolic syndrome, early kidney injury, or early cardiovascular dysfunction, respectively. Specific criteria include hypertension, diabetes, obesity, eGFR, and urinary albumin, among others. CKM stage 4: Clinical cardiovascular disease, which includes coronary heart disease, heart failure, stroke, peripheral arterial disease, and atrial fibrillation, all occurring in the context of CKM. The detailed criteria are provided in Table S1.

### 2.4. Assessment of Frailty

Frailty, serving as the outcome variable, was identified by a frailty index threshold of ≥ 0.21 [21]. This index, initially developed by Sabbah et al. according to Searle et al.′s methodology [22, 23], consists of 49 diagnostic criteria. These criteria cover a broad spectrum of frailty‐related domains, such as cognitive ability, depressive symptoms, daily functioning, physical capability, chronic disease presence, general health, healthcare usage, and laboratory test findings. Table S2 lists the specific items in detail. Each criterion is assigned a score from 0 to 1 according to its severity, and the overall frailty index for an individual is determined by dividing the total sum of their item scores by the number of criteria evaluated. To maintain diagnostic reliability, only participants who answered at least 80% of the items were included in the analysis.

To assess the robustness of our chosen threshold and validate the consistency of our findings, we conducted a sensitivity analysis using an alternative frailty index cutoff of ≥ 0.25. This higher threshold represents a more conservative approach to frailty identification and has been used in previous studies to define moderate to severe frailty states. The sensitivity analysis was performed using the same machine learning algorithms and evaluation metrics to ensure comparability with the primary analysis.

### 2.5. Covariates

To control for confounding effects, multiple demographic and clinical characteristics were included in the analysis. Demographic characteristics: age, sex, race/ethnicity, education level, income (poverty income ratio [PIR]), and marital status. Lifestyle factors: smoking status (never smoker, former smoker, and current smoker) and alcohol consumption (never, former, mild drinking, moderate drinking, and heavy drinking). Anthropometric measurement: BMI. Medical history: diabetes and hypertension. Medication use: antihypertensive drugs, antidiabetic drugs, and lipid‐lowering agents. Laboratory measurements: complete blood count: neutrophils, lymphocytes, monocytes, red blood cells, hemoglobin, and platelet count; liver and kidney function: serum creatinine, UA, BUN, albumin, ALT, and AST; lipid profile: TC, HDL, LDL, and TG; glycemic metabolism: FPG and HbA1c; renal function: eGFR.

### 2.6. Statistical Analysis

Data analyses were performed using R software (Version 4.3.2, https://www.r-project.org/) and SPSS 26.0. In accordance with NHANES guidelines, all analyses incorporated complex sampling design and sample weights to ensure nationally representative estimates. For continuous variables included in the analysis, multicollinearity was assessed, and all VIF were found to be less than 5 (Table S3), indicating no significant multicollinearity. Additionally, LASSO regression was used to select features associated with frailty risk among middle‐aged and older adults with CKM Stages 0–3 for subsequent analyses. To compare general characteristics between frail and nonfrail groups in this population, one‐way analysis of variance was used for continuous variables, and the Pearson chi‐square test was applied for categorical variables. A two‐sided p value < 0.05 was considered statistically significant.

To evaluate potential selection bias resulting from the exclusion of participants with missing data, we conducted a comprehensive comparison of baseline characteristics between the total eligible population before exclusions (*n* = 9042) and the final analytical sample (*n* = 6749). This comparison included demographic variables (age, sex, and race/ethnicity), socioeconomic factors (education level and PIR), clinical characteristics (BMI, blood pressure, and diabetes status), and laboratory measurements that were available in both groups. Chi‐square tests were used for categorical variables, and independent *t*‐tests were employed for continuous variables. Missing data patterns were analyzed to identify systematic differences in data availability across different population subgroups. This analysis is aimed at assessing whether the excluded participants represented a random subset of the eligible population or if specific groups were systematically underrepresented in our final analytical cohort.

In this study, the dataset was randomly divided into a training set and a testing set at a ratio of 7:3. The training set was used for model parameter estimation and fitting, whereas the testing set was used to evaluate model performance. Five machine learning methods were applied: LR, LightGBM, XGBoost, RF, and DT, to predict frailty risk among middle‐aged and older adults with CKM Stages 0–3. To address class imbalance, the synthetic minority over‐sampling technique was used to generate synthetic samples for the minority class, thereby improving model performance [24]. In addition, 10‐fold cross‐validation with repeated iterations was employed to enhance the models’ generalizability and prevent overfitting. Table S4 summarizes the optimal hyperparameters for each model, which were determined by systematic grid search across parameter combinations. Model performance was comprehensively evaluated using accuracy, AUC, precision, recall, and F1 score.

We used the SHAP algorithm to calculate SHAP values for each variable, allowing us to identify the most important features. SHAP is an interpretability method based on game theory that provides a unified explanatory framework for machine learning models, enabling interpretation of complex black‐box models. Each feature has an associated SHAP value for every sample: A positive value indicates that the feature increases the predicted probability, whereas a negative value suggests it decreases the predicted probability. By calculating the weighted average of SHAP values across all features, we can quantify the overall impact of each variable on the model′s predictions. Finally, we conducted an in‐depth analysis of the first six important variables and developed a simple online predictive model using Shinyapps.io that gives health care practitioners easy access to it at any time.

To validate the robustness of our chosen frailty index threshold of ≥ 0.21, we performed a comprehensive sensitivity analysis using an alternative cutoff of ≥ 0.25. The same machine learning pipeline was applied, including LASSO feature selection, model development using the five algorithms (LR, LightGBM, XGBoost, RF, and DT), and performance evaluation using identical metrics. This analysis is aimed at assessing whether the predictive performance and feature importance rankings remained consistent across different frailty definitions, thereby strengthening the reliability of our conclusions.

## 3. Results

### 3.1. Basic Characteristics of Study Population

A total of 6749 middle‐aged and older adults with CKM Stages 0–3 were included in this study, among whom 1427 (21.14%) were classified as frail. The mean age was 56.55 (0.20) years, and 46.87% were male. Compared with nonfrail individuals, frail participants were more likely to be older, non‐Hispanic White women, with lower educational attainment and a higher divorce rate. The prevalence of hypertension, diabetes, and the use of antihypertensive, antidiabetic, and lipid‐lowering medications was also higher among frail participants. Detailed baseline characteristics are shown in Table 1.

**Table 1:** Frailty patients versus nonfrailty patients general clinical characteristics.

Variable	Total	Nonfrailty	Frailty	p value
**Age, mean (SE)**	56.55 (0.20)	56.08 (0.22)	58.66 (0.40)	< 0.001
**PIR, mean (SE)**	3.27 (0.05)	3.44 (0.04)	2.51 (0.08)	< 0.001
**Creatinine, mean (SE)**	78.20 (0.55)	76.94 (0.38)	83.92 (2.63)	0.010
**UA, mean (SE)**	327.06 (1.57)	325.45 (1.61)	334.37 (3.78)	0.020
**BUN, mean (SE)**	5.08 (0.04)	4.99 (0.04)	5.48 (0.09)	< 0.001
**eGFR, mean (SE)**	87.55 (0.38)	88.22 (0.39)	84.52 (0.87)	< 0.001
**TG, mean (SE)**	1.37 (0.02)	1.33 (0.01)	1.54 (0.04)	< 0.001
**TC, mean (SE)**	5.18 (0.02)	5.21 (0.02)	5.06 (0.05)	0.010
**HDL, mean (SE)**	1.45 (0.01)	1.46 (0.01)	1.38 (0.02)	< 0.001
**LDL, mean (SE)**	3.10 (0.02)	3.13 (0.02)	2.97 (0.04)	< 0.001
**BMI, mean (SE)**	29.24 (0.12)	28.72 (0.13)	31.61 (0.29)	< 0.001
**RBC, mean (SE)**	4.69 (0.01)	4.73 (0.01)	4.53 (0.02)	< 0.001
**Hemoglobin, mean (SE)**	14.32 (0.03)	14.44 (0.03)	13.75 (0.06)	< 0.001
**Platelet, mean (SE)**	239.05 (1.24)	236.39 (1.30)	251.15 (2.42)	< 0.001
**Albumin, mean (SE)**	4.21 (0.01)	4.23 (0.01)	4.09 (0.01)	< 0.001
**Lymphocyte, mean (SE)**	1.92 (0.01)	1.89 (0.01)	2.07 (0.03)	< 0.001
**Monocyte, mean (SE)**	0.54 (0.00)	0.53 (0.00)	0.57 (0.01)	< 0.001
**Neutrophils, mean (SE)**	3.91 (0.04)	3.80 (0.04)	4.43 (0.07)	< 0.001
**FPG, mean (SE)**	6.08 (0.03)	5.96 (0.03)	6.64 (0.07)	< 0.001
**HbA1c, mean (SE)**	5.76 (0.02)	5.67 (0.01)	6.15 (0.04)	< 0.001
**ALT, mean (SE)**	25.12 (0.27)	25.13 (0.32)	25.06 (0.69)	0.930
**AST, mean (SE)**	25.53 (0.26)	25.35 (0.28)	26.39 (0.86)	0.260
**Sex, %(SE)**				< 0.001
Female	53.13 (0.02)	50.29 (0.74)	66.07 (1.75)	
Male	46.87 (0.02)	49.71 (0.74)	33.93 (1.75)	
**Race, %(SE)**				< 0.001
Mexican American	6.28 (0.01)	6.23 (0.65)	6.50 (0.85)	
Non‐Hispanic Black	8.68 (0.01)	7.83 (0.65)	12.55 (1.25)	
Non‐Hispanic White	74.16 (0.04)	75.25 (1.42)	69.15 (1.97)	
Others	10.89 (0.00)	10.69 (1.01)	11.80 (1.69)	
**Marital, %(SE)**				< 0.001
Divorced	14.76 (0.01)	14.08 (0.82)	17.88 (1.42)	
Married	63.77 (0.03)	66.53 (1.16)	51.17 (2.25)	
Never married	7.68 (0.00)	7.28 (0.51)	9.46 (1.19)	
Others	13.79 (0.01)	12.10 (0.61)	21.48 (1.38)	
**Education, %(SE)**				< 0.001
High school or equivalent	22.49 (0.02)	21.40 (1.57)	27.46 (2.85)	
Less than high school	14.73 (0.01)	12.98 (0.80)	22.75 (1.33)	
Some college or above	62.77 (0.03)	65.62 (1.38)	49.79 (2.20)	
**Smoke, %(SE)**				< 0.001
Former	29.44 (0.02)	29.43 (1.02)	29.48 (1.82)	
Never	53.84 (0.02)	56.08 (1.04)	43.60 (2.11)	
Now	16.72 (0.01)	14.48 (0.76)	26.93 (2.10)	
**Alcohol, %(SE)**				< 0.001
Former	17.20 (0.01)	15.04 (0.74)	27.09 (1.57)	
Heavy	15.32 (0.01)	15.33 (0.69)	15.26 (1.56)	
Mild	40.64 (0.02)	42.92 (1.15)	30.27 (2.02)	
Moderate	16.65 (0.01)	17.09 (0.75)	14.65 (1.46)	
Never	10.18 (0.01)	9.62 (0.64)	12.73 (1.15)	
**Hypertension, %(SE)**				< 0.001
No	52.94 (0.02)	58.14 (1.02)	29.26 (1.55)	
Yes	47.06 (0.02)	41.86 (1.02)	70.74 (1.55)	
**Diabetes, %(SE)**				<0.001
Borderline	20.78 (0.01)	20.82 (0.70)	20.60 (1.44)	
No	59.23 (0.02)	63.26 (0.93)	40.88 (1.84)	
Yes	19.99 (0.01)	15.92 (0.74)	38.52 (1.50)	
**Antidiabetic, %(SE)**				< 0.001
No	88.65 (0.03)	92.06 (0.54)	73.10 (1.54)	
Yes	11.35 (0.01)	7.94 (0.54)	26.90 (1.54)	
**Antihypertension, %(SE)**				< 0.001
No	63.65 (0.02)	69.39 (1.00)	37.45 (1.76)	
Yes	36.35 (0.01)	30.61 (1.00)	62.55 (1.76)	
**Antihyperlipidemic, %(SE)**				< 0.001
No	76.00 (0.03)	79.36 (0.84)	60.(1.75)	
Yes	24.00 (0.01)	20.64 (0.84)	39.(1.75)	

*Note:* Date are presented as mean (SE) or *n* (%).

Abbreviations: ALT, alanine aminotransferase; AST, aspartate aminotransferase; BMI, body mass index; BUN, blood urea nitrogen; eGFR, estimated glomerular filtration rate; FPG, fasting plasma glucose; HbA1c, glycosylated hemoglobin; HDL, high density lipoprotein; LDL, low density lipoprotein; PIR, poverty income ratio; RBC, red blood cell; TC, total cholesterol; TG, triglyceride; UA, uric acid.

### 3.2. Assessment of Selection Bias

The comparison of characteristics between participants before and after exclusion for missing data revealed several important differences that suggest the presence of selection bias (Table S5). Among the 2293 excluded participants, there was a higher proportion of individuals aged 65 years and older (42.3% vs. 31.7%, *p* < 0.001), suggesting that older adults were more likely to have incomplete data. Excluded participants were also more likely to be from racial/ethnic minority groups, with a higher percentage of non‐Hispanic Black (12.8% vs. 8.7%, *p* < 0.001) and Mexican American participants (8.9% vs. 6.3%, *p* < 0.001) compared with those included in the final analysis.

Socioeconomic disparities were evident in the missing data patterns, with excluded participants having significantly lower PIRs (mean PIR: 2.84 vs. 3.27, *p* < 0.001) and lower educational attainment (28.7% vs. 14.7% with less than high school education, *p* < 0.001). Clinical characteristics also differed significantly between groups. Excluded participants had higher rates of diabetes (27.4% vs. 20.0%, *p* < 0.001), hypertension (52.1% vs. 47.1%, *p* < 0.001), and were more likely to be using antihypertensive medications (41.3% vs. 36.4%, *p* < 0.01). Additionally, laboratory data availability was particularly problematic among participants with chronic kidney disease, with 34.2% of those with eGFR < 60 mL/min/1.73m^2^ having incomplete laboratory profiles compared with 18.7% of those with normal kidney function (*p* < 0.001).

The missing data analysis revealed that frailty‐related variables were more likely to be incomplete among participants who would have been classified as high‐risk based on available data. Specifically, participants with incomplete functional assessment data had higher rates of chronic diseases and medication use, suggesting that our final analytical sample may underrepresent the most vulnerable populations. These findings indicate that our predictive model was developed on a population that may be healthier, younger, and of higher socioeconomic status than the broader CKM population, potentially limiting the generalizability of our results to the most at‐risk individuals.

### 3.3. Important Factors in Screening for Risk of Frailty in Middle‐Aged and Older Patients With CKM Stages 0–3 by LASSO Profile

To select the most suitable variables, LASSO regression was applied. As shown in Figure 2a, with an increase in log(*λ*), the coefficients in the model gradually approach zero, indicating that the independent variables are eliminated one by one. In Figure 2b, it can be observed that all variables are excluded at lambda.1se, leaving no coefficients in the model; whereas at lambda.min, the LASSO regression model retains 21 variables and achieves the lowest prediction error. Based on 10‐fold cross‐validation, LASSO ultimately identified sex, marital, smoke, alcohol, hypertension, diabetes, antidiabetic, antihypertension, antihyperlipidemic, PIR, BMI, monocyte, neutrophils, RBC, hemoglobin, FPG, AST, albumin, creatinine, BUN, and TG as the optimal independent variables in the training set.

Figure 2Presentation of the results of the LASSO regression analysis. (a) LASSO regression model screening variable trajectories. (b) LASSO regression model factor selection: Left dashed line represents the optimal lambda value (lambda.min), whereas the right dashed line marks the lambda value within one standard error of the optimal (lambda.1se).

**Figure:**
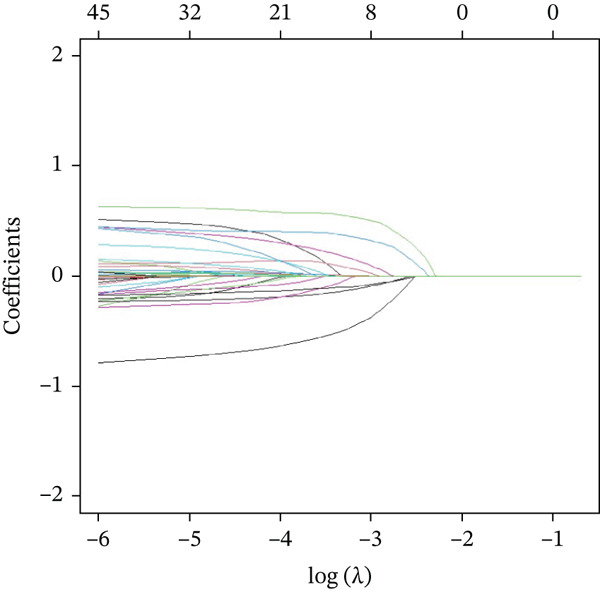
(a)

**Figure:**
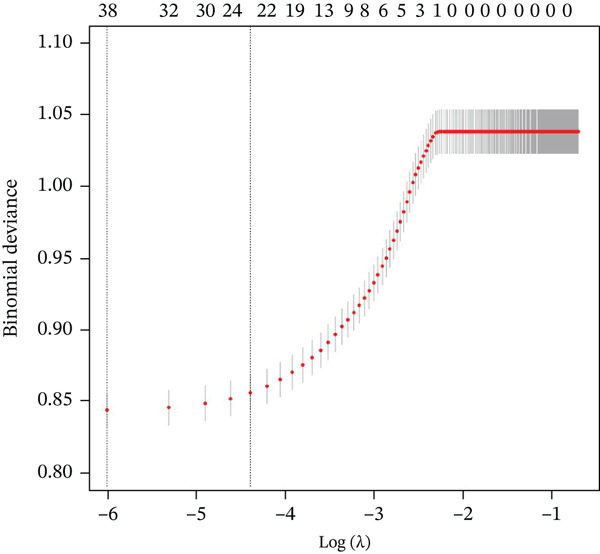
(b)

### 3.4. Model Development and Validation

Five machine learning models LR, LightGBM, XGBoost, RF, and DT were constructed for this study. The data were randomly divided into training and testing sets at a ratio of 7:3. Figure 3 displays the ROC curves of these five ML models in both the training and validation sets. Among them, the ROC curve of the RF model was closest to the upper left corner in the training set, indicating the best predictive performance. Figure 4 compares the accuracy, AUC, precision, recall, and F1 score across models, demonstrating that the RF model performed optimally in the training set and achieved satisfactory accuracy in the testing set. Furthermore, decision curve analysis and calibration curves (Figures S1 and S2) also confirmed the clinical utility of the RF model. Therefore, the RF model was ultimately selected for further analysis.

Figure 3ROC analysis of machine learning model; LightGBM: light gradient boosting machine; (a) training set (b) validation set; DT: decision tree, XGBoost: extreme gradient boosting, LR: logistic regression, RF: random forest.

**Figure:**
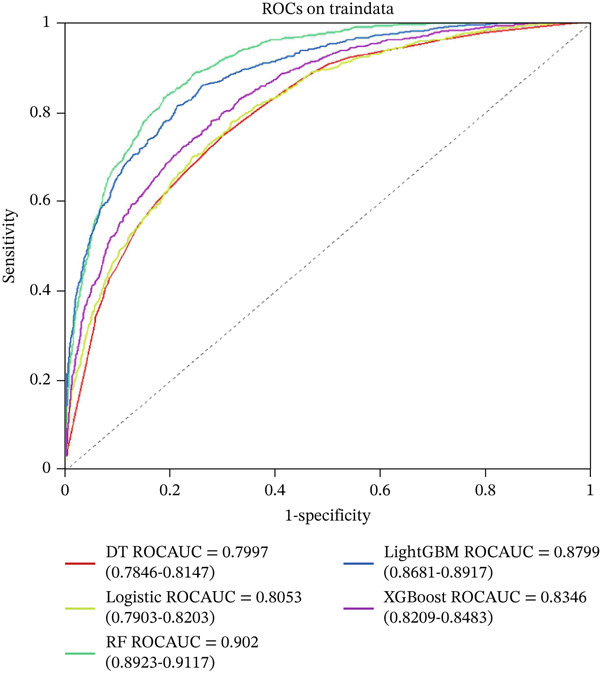
(a)

**Figure:**
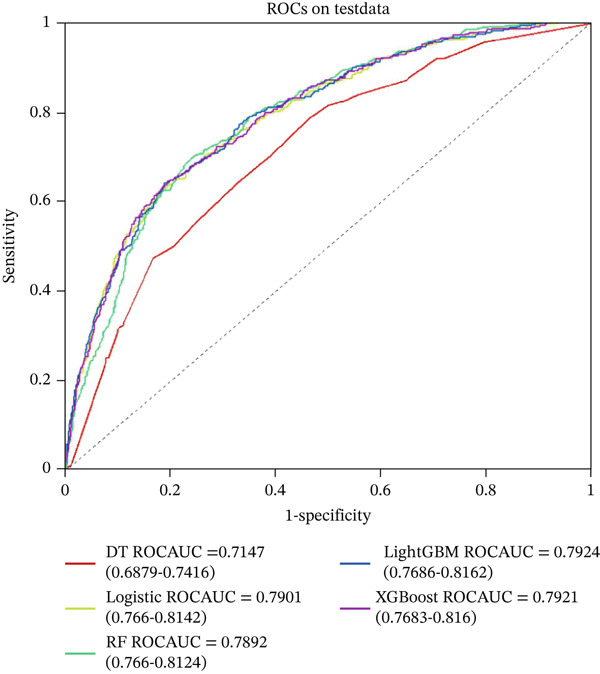
(b)

Figure 4Performance comparison of different machine learning models on the training (a) and test (b) sets across multiple evaluation metrics. The heatmaps display the performance of DT: decision tree, XGBoost: extreme gradient boosting, LR: logistic regression, RF: random forest. Each cell represents the value of a specific evaluation metric, including accuracy, balanced accuracy, F1 score, J‐index, kappa, Matthew′s correlation coefficient (MCC), positive predictive value (PPV), negative predictive value (NPV), precision, recall, ROC AUC, sensitivity (sens), and specificity (spec). Higher values are indicated by red, whereas lower values are represented by green, showing the model′s effectiveness in both training and test sets.

**Figure:**
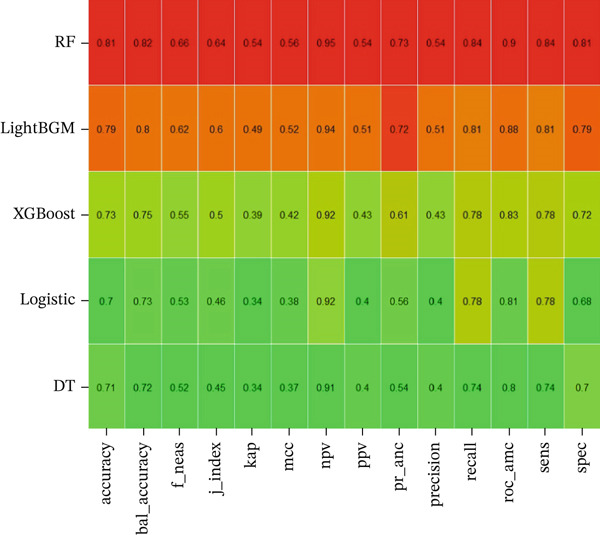
(a)

**Figure:**
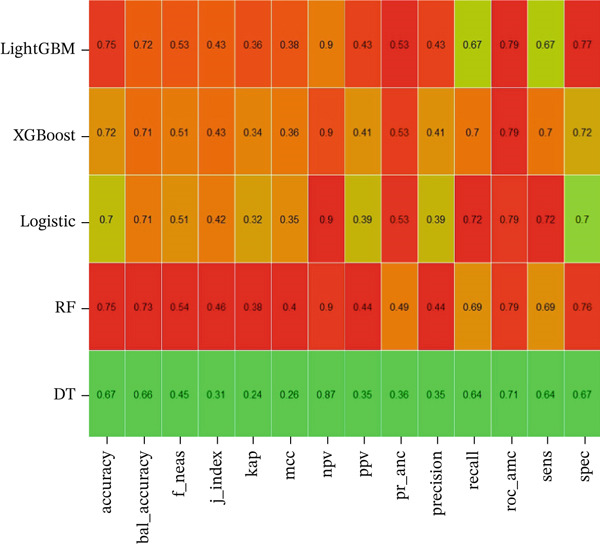
(b)

### 3.5. Sensitivity Analysis Results

The sensitivity analysis using a frailty index threshold of ≥ 0.25 identified 892 participants (13.22%) as frail, compared with 1427 participants (21.14%) with the ≥ 0.21 threshold. Despite the reduced prevalence, the machine learning models maintained robust predictive performance. The random forest model continued to demonstrate superior performance with the ≥ 0.25 threshold, achieving an AUC of 0.88 (95% CI: 0.85–0.91), accuracy of 0.85 (95% CI: 0.82–0.88), precision of 0.58 (95% CI: 0.52–0.64), recall of 0.79 (95% CI: 0.73–0.85), and F1 score of 0.67 (95% CI: 0.62–0.72) in the testing set. Importantly, the feature importance rankings remained largely consistent between the two thresholds. PIR maintained its position as the most influential predictor (SHAP value: 0.146), followed by antihypertensive medication use (SHAP value: 0.128), hemoglobin (SHAP value: 0.121), red blood cell count (SHAP value: 0.115), albumin (SHAP value: 0.109), and diabetes status (SHAP value: 0.103). The correlation coefficient between feature importance rankings of the two analyses was 0.94 (p < 0.001), indicating excellent consistency. Table S6 presents a detailed comparison of model performance metrics between the two thresholds. The sensitivity analysis confirmed that our machine learning approach is robust across different frailty definitions, with only minimal variations in predictive accuracy while maintaining the same hierarchical importance of risk factors.

### 3.6. Model Interpretation

We performed SHAP analysis on the RF model to assess the importance of each feature and its impact on prediction outcomes (Figure 5a,b). The results showed that PIR was the most critical variable, with the highest SHAP value, playing a dominant role in risk prediction among middle‐aged and older adults with CKM Stages 0–3. In addition, the use of antihypertensive medications, hemoglobin, red blood cell count, albumin, and the presence of diabetes also had significant effects on patient risk.

Figure 5SHAP feature importance analysis for random forest model predicting frailty risk in ckm patients. (a) Feature importance ranking plot: Horizontal bar chart displaying the mean absolute SHAP values for the top predictive features, ranked in descending order of importance. Each bar represents the average impact magnitude of a feature on model predictions across all samples. PIR, poverty income ratio; RBC, red blood cell count; FPG, fasting plasma glucose; BUN, blood urea nitrogen; TG, triglycerides; AST, aspartate aminotransferase. (b) SHAP summary plot: Dot plot showing the distribution of SHAP values for each feature across all samples. Each dot represents an individual patient. The *x*‐axis shows SHAP values (feature impact on prediction), where positive values increase frailty risk prediction and negative values decrease it. The *y*‐axis lists features ranked by importance (top to bottom). Color coding: Red dots indicate high feature values, blue dots indicate low feature values, and color intensity reflects the magnitude of the feature value. Dot position along the *x*‐axis indicates the direction and magnitude of each feature′s contribution to the individual prediction. Wide distribution of dots indicates high feature variability, whereas clustering suggests consistent feature effects across patients.

**Figure:**
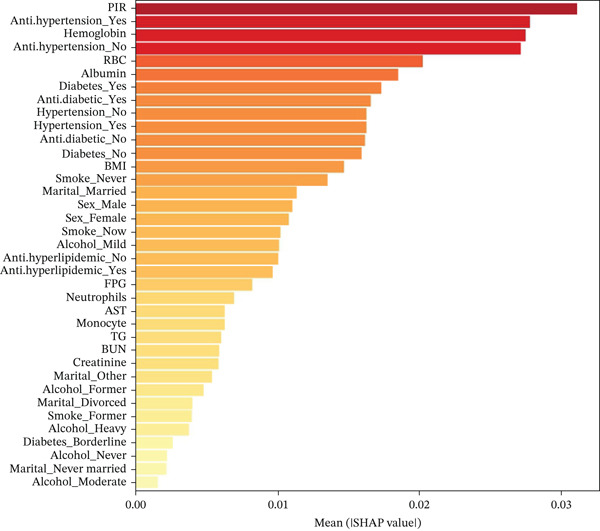
(a)

**Figure:**
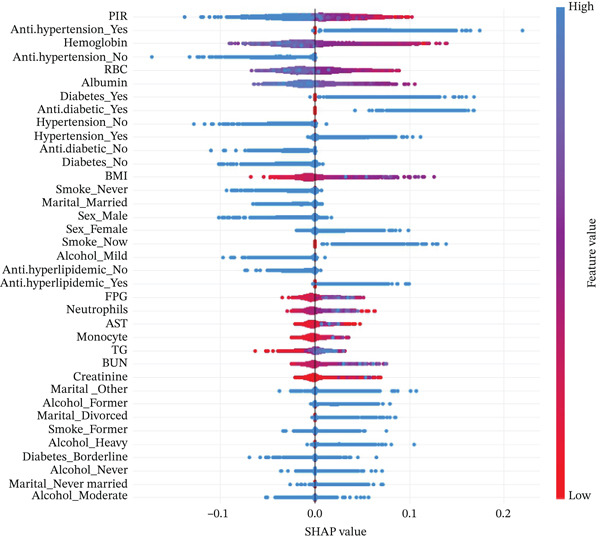
(b)

Figure 6 presents the SHAP dependence plots for both continuous and categorical variables in the RF model. As shown in Figure 6a, patients taking antihypertensive medications and those with diabetes had higher SHAP values, indicating a greater risk of frailty among middle‐aged and older adults with CKM Stages 0–3 in these groups. Figure 6b demonstrates a negative correlation between PIR and SHAP values, suggesting that higher PIR is associated with a lower risk of frailty. Additionally, as hemoglobin and RBC levels increase, their SHAP values decrease initially but begin to rise again after surpassing a certain threshold. Figure 6b also shows that albumin exerts a negative influence on the model within the range of 3–4.5, but beyond this range, its impact becomes positive.

Figure 6SHAP dependence plots illustrating nonlinear relationships between key features and frailty risk. (a) Categorical variables dependence analysis: Bar plots showing SHAP values (*y*‐axis) for categorical predictors. Antihypertensive medication use: Blue bars represent patients not using antihypertensive medications (value = 0), orange bars represent patients using antihypertensive medications (value = 1). Higher SHAP values indicate increased frailty risk. Diabetes status shows SHAP value distributions across diabetes categories, where higher values correspond to increased frailty risk prediction. (b) Continuous variables dependence analysis: Scatter plots displaying the relationship between feature values (*x*‐axis) and their corresponding SHAP values (*y*‐axis) for continuous predictors. Color coding: Points are colored by feature interaction effects, typically representing the value of another correlated feature to show interaction patterns. PIR (poverty income ratio) shows inverse relationship where higher PIR values (better economic status) correspond to lower SHAP values (reduced frailty risk). Hemoglobin demonstrates U‐shaped relationship with optimal range around 12.5–15.0 g/dL, where both low (< 12 g/dL) and high (> 16 g/dL) values increase frailty risk. RBC (red blood cell count): Similar nonlinear pattern with optimal range approximately 4.2–4.8 × 10^12^/L. Albumin: Shows protective effects within 3.0–4.5 g/dL range, with increased risk at both extremes, indicating optimal rather than maximal values are preferred. Abbreviations: PIR, poverty income ratio; RBC, red blood cell count.

**Figure:**
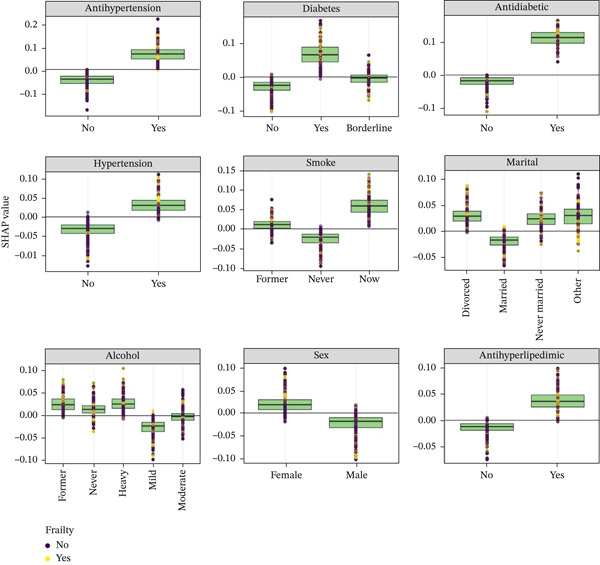
(a)

**Figure:**
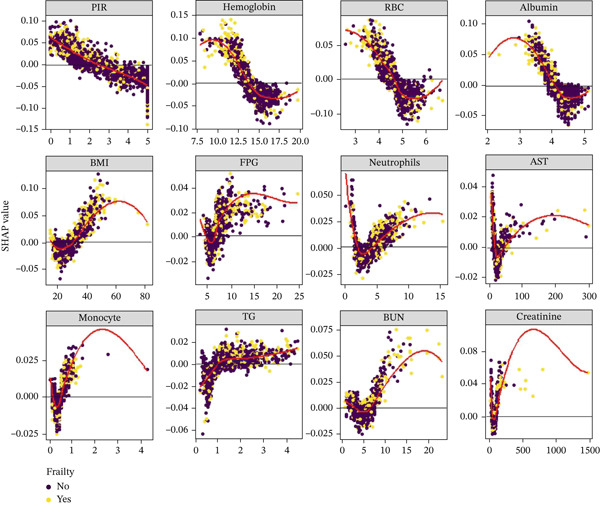
(b)

The waterfall plot (Figure 7a) illustrates the specific risk prediction of frailty for an individual middle‐aged or older patient with CKM Stages 0–3. The results show that diabetes, use of antihypertensive medications, and use of antidiabetic drugs significantly increased the SHAP value (0.744), which corresponded with the actual occurrence of frailty in this patient. Figure 7b presents the prediction for another CKM Stages 0–3 patient who did not develop frailty; higher PIR and absence of hypertension contributed to a lower SHAP value (0.0918), which was consistent with the true outcome. The force plots (Figure 7c,d) visually display the SHAP values for each feature in determining the final prediction; yellow bars represent risk factors, and purple bars represent protective factors, with longer bars indicating greater influence. These visualizations provide a more nuanced understanding of individual risk factors and offer important insights for applying the prediction model in clinical practice.

Figure 7Individual patient risk prediction examples using SHAP analysis for CKM Stages 0–3 patients. (a) Waterfall plot—high‐risk patient (frail) shows step‐by‐step contribution of each feature to the final prediction for a patient who developed frailty. The plot starts from the base value (population average risk) and shows how each feature incrementally increases (+) or decreases (−) the prediction probability. Red bars represent risk‐increasing factors and blue bars represent protective factors. Bar length indicates the magnitude of each feature′s contribution. Final prediction value: 0.744 (high frailty risk), which correctly predicted the actual frailty outcome. Key contributors include diabetes (+0.168), antihypertensive medication use (+0.141), and antidiabetic medication use (+0.125). (b) Waterfall plot—low‐risk patient (nonfrail): similar analysis for a patient who remained nonfrail. Final prediction value: 0.0918 (low frailty risk), correctly predicting the actual outcome. Higher PIR and absence of hypertension were major protective factors. (c) Force plot—high‐risk patient: Horizontal bar visualization showing feature contributions for the frail patient. Yellow/orange bars extending right represent risk factors pushing prediction toward positive outcome and blue/purple bars extending left represent protective factors. Bar width corresponds to feature importance magnitude. The final prediction (0.744) is positioned on the scale from 0 (low risk) to 1 (high risk). (d) Force plot—low‐risk patient: Same visualization format for the nonfrail patient, showing how protective factors (primarily higher PIR and absence of comorbidities) result in low risk prediction (0.0918). Interpretation: These individual‐level analyses demonstrate model transparency by showing exactly how each patient′s characteristics contribute to their personalized frailty risk assessment, enabling clinicians to understand the reasoning behind each prediction and identify modifiable risk factors for targeted interventions.

**Figure:**
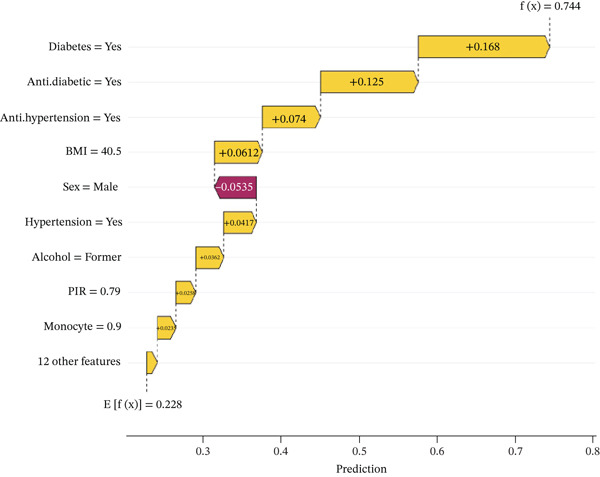
(a)

**Figure:**
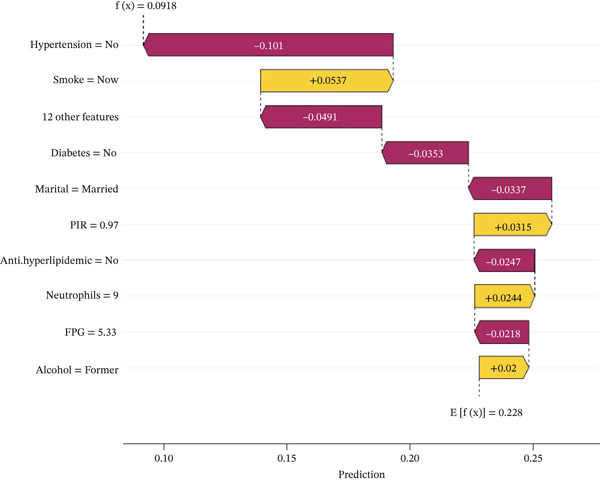
(b)

**Figure:**
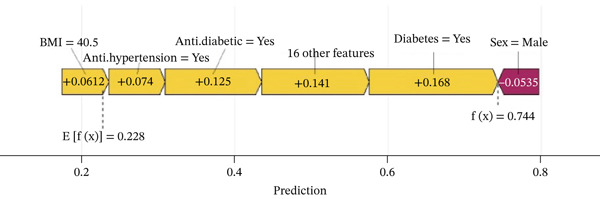
(c)

**Figure:**
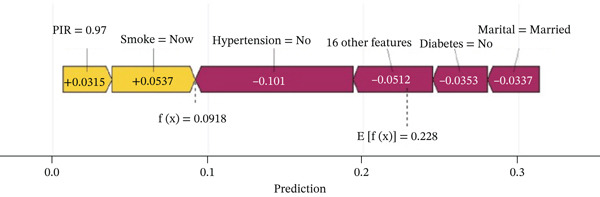
(d)

### 3.7. Web Calculator Implementation

**Figure 8:**
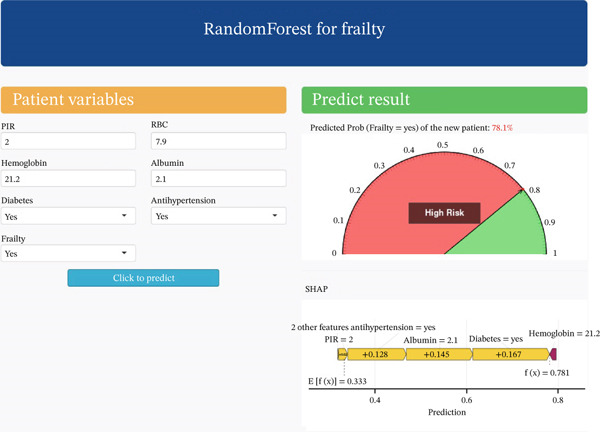
Interactive interface of random forest‐based frailty risk prediction model with SHAP explainability analysis. Patient variables input module: The left panel provides input fields for key physiological indicators, including poverty income ratio (PIR = 2), Red blood cell count (RBC = 7.9), hemoglobin level (21.2 g/dL), serum albumin (2.1 g/dL), and binary status variables for diabetes and antihypertension treatment (both set to “Yes”). Prediction result visualization: The semicircular gauge in the upper right section intuitively displays the patient′s frailty risk probability at 78.1%. The red area represents the high‐risk range, whereas the green area indicates the low‐risk range, providing clear risk stratification assessment. SHAP value explanation plot: The feature contribution analysis chart at the bottom, based on SHAP (SHapley Additive exPlanations) methodology, quantifies the specific impact of each input feature on the final prediction outcome. The plot shows the model′s baseline expected value *E*[*f*(*x*)] = 0.333 and the current patient′s predicted value *f*(*x*) = 0.781. Feature contributions are ranked by impact magnitude: Diabetes combined with hemoglobin abnormality (+0.167), hypoalbuminemia (+0.145), and poverty status (PIR = 2, +0.128) all serve as positive risk factors, significantly elevating the frailty probability prediction.

We developed a deployable web platform to analyze the importance of features through the SHAP algorithm to construct a calculator for the top six variables for frailty risk in CKM patients (web address: https://cvdshiny.shinyapps.io/Frailty/). This platform provides a convenient online tool for assessing the risk of frailty in CKM patients. Users simply enter the clinical feature data into a designated text box to easily obtain the desired prediction (Figure 8).

## 4. Discussion

Based on NHANES data, this study developed a machine learning‐based prediction model for frailty risk among middle‐aged and older adults with CKM Stages 0–3. Through feature selection using LASSO regression and comparison of multiple machine learning algorithms, we found that the RF model performed best. SHAP analysis was then used to identify key predictors associated with frailty risk in CKM patients.

Our results indicate that the PIR is the most important factor in predicting frailty risk among CKM patients, which is consistent with previous research on the relationship between socioeconomic status and health outcomes [25]. Socioeconomic status not only directly affects access to healthcare and health‐related behaviors, but may also influence frailty through pathways such as chronic stress and inflammation [26]. In a large Dutch cohort study, Hoogendijk et al. also found that lower income and educational levels were significantly associated with increased frailty risk [27]. These findings highlight the importance of considering socioeconomic factors in the management of CKM patients and suggest that such factors should be incorporated into risk assessment tools.

Use of antihypertensive medications, identified as the second most important predictor, shows a strong association with frailty risk, though the cross‐sectional nature of our study precludes determining causality. This association may reflect multiple factors: the severity of underlying cardiovascular disease, intensity of treatment required, or potentially the effects of medication themselves. It is important to note that medication use may be a marker of disease severity rather than a causal factor for frailty development. The relationship between cardiovascular medications and frailty is complex and likely bidirectional: although these medications may reduce organ damage by controlling blood pressure, polypharmacy, adverse drug reactions, and potential hypotension may also contribute to frailty risk [28]. Palmer et al., in a systematic review, pointed out that the association between antihypertensive treatment and frailty among elderly hypertensive patients remains controversial, emphasizing the importance of individualized treatment strategies [29]. Our findings identify this association but cannot establish whether medication use contributes to frailty risk or simply reflects more advanced disease requiring pharmaceutical intervention. Prospective studies are needed to elucidate the causal relationships between antihypertensive therapy and frailty outcomes in CKM patients.

Hematological indicators (such as hemoglobin and red blood cell count) and nutritional status markers (such as albumin) also demonstrated significant predictive value in our model. Anemia is common among patients with chronic kidney disease and heart failure and has been shown to be closely associated with frailty [30]. Nishiguchi et al. found that hemoglobin levels are positively correlated with muscle mass, grip strength, and physical function in older adults [31].

Our SHAP dependence plots revealed a nonlinear relationship between hemoglobin and frailty risk; both low and excessively high hemoglobin levels may increase frailty risk, a finding that goes beyond the limitations of traditional linear models in capturing such complex associations. This U‐shaped relationship has profound clinical implications for CKM patient management. The increased risk at low hemoglobin levels (< 12 g/dL) reflects well‐established anemia‐related functional decline, whereas the unexpected elevated risk at high levels (> 15–16 g/dL) may result from increased blood viscosity, enhanced thrombotic potential, and heightened oxidative stress—mechanisms particularly relevant in CKM patients with existing cardiovascular and renal compromise. This finding suggests optimal hemoglobin targets of 12.5–15.0 g/dL for minimizing frailty risk, challenging traditional approaches that focus solely on correcting anemia without considering upper safety limits.

Similarly, albumin demonstrated a nonlinear relationship with optimal protective effects occurring within the 3.0–4.5 g/dL range. This finding has important clinical implications beyond simple malnutrition screening [32]. Although hypoalbuminemia clearly indicates protein‐energy malnutrition and chronic inflammation, the increased frailty risk observed with elevated albumin levels (> 4.5 g/dL) may reflect dehydration, hemoconcentration, or underlying pathological processes that paradoxically indicate poor clinical status despite seemingly favorable laboratory values. For CKM patients, this suggests the need for comprehensive assessment rather than assuming optimal health based on high albumin levels alone. These nonlinear relationships support a precision medicine approach that targets optimal biomarker ranges rather than simply correcting abnormal values, enabling more sophisticated risk stratification and therapeutic decision‐making in complex CKM patients.

Diabetes, as a significant component of CKM syndrome, was identified as an important predictor of frailty in our model. This finding is consistent with numerous studies; for example, Sinclair et al. demonstrated that diabetes contributes to frailty development and decline in muscle function through multiple mechanisms, including insulin resistance, microvascular complications, and chronic inflammation [10]. Notably, our study also found that the use of antidiabetic medications is strongly associated with frailty risk. Given the cross‐sectional design, this association most likely reflects disease severity and duration rather than indicating that antidiabetic medications causally contribute to frailty. However, this finding also highlights the complex potential interactions between glycemic control therapies and frailty development that warrant further investigation in longitudinal studies [33]. The observed association may indicate that patients requiring pharmacological diabetes management represent a higher risk population due to more advanced metabolic dysfunction rather than medication effects per se.

Through individual‐level waterfall and force plot analyses, we illustrated how the model integrates multiple risk factors to provide personalized predictions. This interpretable approach not only enhances model transparency but also offers practical guidance for clinical decision‐making. For instance, in patients with both diabetes and hypertension who are on multiple medications, the model predicts a significantly increased risk of frailty, which aligns with actual outcomes. Conversely, in patients with favorable socioeconomic status and no hypertension, the model accurately predicts a lower frailty risk. Such individualized risk predictions can help clinicians identify high‐risk patients and implement early interventions [34].

Importantly, given the cross‐sectional nature of our study, all identified relationships represent associations rather than causal relationships. This distinction is particularly critical when interpreting medication‐related predictors, as these associations may reflect disease severity markers, treatment intensity indicators, or confounding by indication rather than direct causal effects of medications on frailty development. The temporal sequence of events cannot be established from cross‐sectional data, and reverse causation remains a possibility—frail individuals may be more likely to receive certain medications due to their higher disease burden or may be prescribed specific treatments as a consequence of their frail status. Future longitudinal studies are essential to elucidate the causal pathways underlying these observed associations and to inform evidence‐based clinical decision‐making.

The sensitivity analysis using an alternative frailty index threshold of ≥ 0.25 provided important validation of our findings. The consistency of model performance and feature importance rankings across different thresholds strengthens confidence in our results and suggests that the identified predictors are genuinely associated with frailty risk rather than being artifacts of the specific cutoff chosen. This is particularly important for clinical application, as it indicates that our model can reliably identify high‐risk individuals regardless of whether a more liberal (≥ 0.21) or conservative (≥ 0.25) approach to frailty definition is adopted. The slight improvement in precision observed with the higher threshold (0.58 vs. 0.54) reflects the expected trade‐off between sensitivity and specificity when using more stringent diagnostic criteria. However, the maintained high recall (0.79 vs. 0.84) suggests that the model continues to identify the vast majority of truly frail individuals even with the more conservative definition. This flexibility in threshold selection allows clinicians to adapt the screening approach based on their specific clinical context and resource availability.

Compared with previous studies, our research possesses several notable strengths. First, we focused on CKM syndrome as a multisystem disease state, taking into account the interactions between different systems rather than treating each disease in isolation. Second, we employed advanced machine learning techniques, particularly the random forest algorithm, which excels at handling nonlinear relationships and interactions between variables [12]. Third, by using SHAP analysis, we not only identified important predictive factors but also elucidated how these variables influence the prediction results, thereby enhancing the interpretability of our model. Lastly, our model was developed using data from NHANES, a nationally representative database, which improves its external validity and generalizability.

However, this study also has several important limitations. First, the cross‐sectional design fundamentally limits our ability to establish causal relationships between predictors and frailty risk. All identified associations, particularly those involving medication use, should be interpreted as correlative rather than causal. The observed relationships may reflect disease severity, treatment intensity, confounding by indication, or reverse causation, where frailty status influences medication prescribing patterns. Second, our analysis of selection bias revealed significant systematic differences between included and excluded participants, with older adults, racial/ethnic minorities, individuals with lower socioeconomic status, and those with more severe chronic conditions being disproportionately excluded due to missing data. This selection bias suggests that our predictive model was developed on a population that may be healthier and of higher socioeconomic status than the broader CKM population, potentially limiting the generalizability of our results to the most vulnerable and highest risk individuals. The underrepresentation of these high‐risk groups may lead to an underestimation of frailty prevalence and could affect the model′s performance when applied to more diverse or disadvantaged populations. Third, although our model included a wide range of clinical and laboratory variables, there may still be important unmeasured factors such as muscle mass, detailed cognitive function assessments, and social support networks that could improve predictive accuracy. Additionally, although NHANES is representative of the civilian, noninstitutionalized US population, it does not include institutionalized populations, which may further contribute to an underestimation of severe frailty prevalence. The systematic exclusion of participants with incomplete data, particularly those with complex medical conditions requiring extensive laboratory monitoring, may have inadvertently excluded individuals most likely to develop frailty, thereby limiting the clinical utility of our model in identifying the highest risk patients. Finally, external validation of the model is necessary to assess its performance across different populations and healthcare settings, particularly those serving more vulnerable populations with higher rates of missing data. Future research should focus on: (1) developing strategies to minimize missing data, particularly among vulnerable populations; (2) employing multiple imputation or other advanced techniques to handle missing data more effectively; (3) validating model performance in independent cohorts that include more representative samples of high‐risk populations; and (4) assessing the clinical implementation and effectiveness of early interventions based on model predictions across diverse healthcare settings.

In summary, the machine learning model developed in this study using NHANES data can effectively predict frailty risk among middle‐aged and older adults with CKM Stages 0–3 and identify key predictors, including socioeconomic status, cardiovascular and metabolic disease status, medication use, as well as hematological and biochemical markers. These findings provide new insights into the complex relationship between CKM and frailty and offer tools for identifying high‐risk patients and implementing early interventions in clinical practice. By integrating multisystem factors for risk prediction, our study supports a holistic and individualized management strategy for CKM patients, with the potential to improve health outcomes in this complex patient population.

**Nomenclature**
CKM: cardiovascular‐kidney‐metabolic syndrome
NHANES: National Health and Nutrition Examination Survey
SHAP: Shapley Additive exPlanations
PIR: poverty income ratio
BMI: body mass index
FPG: fasting plasma glucose
RBC: red blood cell
HbA1c: glycosylated hemoglobin
UA: uric acid
BUN: blood urea nitrogen
TG: triglyceride
TC: total cholesterol
HDL: high density lipoprotein
LDL: low density lipoprotein
eGFR: estimated glomerular filtration rate
ALT: alanine aminotransferase
AST: aspartate aminotransferase
DT: decision tree
XGBoost: extreme gradient boosting
LR: logistic regression
RF: random forest
VIF: variance inflation factors
ROC: receiver operating characteristic curve
AUC: area under curve

## Author Contributions

Wenlong Ding made important contributions to conception of the study, method design, and writing and editing of the manuscript. Zheng Wang and Caoyang Fang were mainly responsible for the data analysis. Qin Cui, Lei Fang, and Fachao Shi reviewed and edited the manuscript to ensure its scientific rigor and clarity, contributing critical input in refining the final draft. Wenlong Ding and Caoyang Fang have contributed equally and are regarded as co‐first authors.

## Funding

No funding was received for this manuscript.

## Ethics Statement

The study was conducted according to the Declaration of Helsinki. All information from the NHANES program is freely available to the public and therefore does not require approval from the Medical Ethics Committee.

## Conflicts of Interest

The authors declare no conflicts of interest.

## Supporting information


**Supporting Information** Additional supporting information can be found online in the Supporting Information section. Table S1: Definition of CKM. Table S2: Items in the 49‐item frailty index and corresponding detailed scoring criteria. Table S3: Baseline characteristics according to HGI quartiles. Table S4: Optimal hyperparameter settings for five models based on 10‐fold cross‐validation and grid search strategy. Table S5: Baseline characteristics comparison: included versus excluded participants due to missing data characteristic included excluded difference *p* value. Table S6: Model performance comparison between different frailty index thresholds. Figure S1: Decision curve analysis of machine learning model; (a) training set, (b) validation set; DT, decision tree; XGBoost, extreme gradient boosting; LR, logistic regression; RF, random forest. Figure S2: Calibration curve of machine learning model; (a) training set, (b) validation set; DT, decision tree; XGBoost, extreme gradient boosting; LR, logistic regression; RF, random forest.

## Data Availability

The data that support the findings of this study are available from the corresponding author upon reasonable request.
