# Supplementary material for: NHANES‐Derived Machine Learning Model for Early Identification of Frailty Risk in CKM: Optimizing Resource Allocation Through Predictive Analytics
Source: Cardiovasc Ther. 2026 Mar 18;2026:7296443. doi: 10.1155/cdr/7296443 (PMC13140421; doi:10.1155/cdr/7296443)
Supplement: Supplementary file 1 — Supporting Information Additional supporting information can be found online in the Supporting Information section. Table S1: Definition of CKM. Table S2: Items in the 49‐item frailty index and corresponding detailed scoring criteria. Table S3: Baseline characteristics according to HGI quartiles. Table S4: Optimal hyperparameter settings for five models based on 10‐fold cross‐validation and grid search strategy. Table S5: Baseline characteristics comparison: included versus excluded participants due to missing data characteristic included excluded difference p value. Table S6: Model performance comparison between different frailty index thresholds. Figure S1: Decision curve analysis of machine learning model; (a) training set, (b) validation set; DT, decision tree; XGBoost, extreme gradient boosting; LR, logistic regression; RF, random forest. Figure S2: Calibration curve of machine learning model; (a) training set, (b) validation set; DT, decision tree; XGBoost, extreme gradient boosting; LR, logistic regression; RF, random forest. [file CDR-2026-7296443-s001.docx]

**NHANES-Derived Machine Learning Model for Early Identification of Frailty Risk in CKM: Optimizing Resource Allocation Through Predictive Analytics**

Wenlong Ding^1,+^,Caoyang Fang^2,+^,Fachao Shi^3^,Lei Fang^4^,Qin Cui^4^,Zheng Wang^5,*^

^1^Department of Cardiology, Xuancheng Hospital Affiliated to Wannan Medical College (Xuancheng People 's Hospital), Xuancheng Anhui 242000, China;

^2^Department of Emergency,The First Affiliated Hospital of USTC,Division of Life Sciences and Medicine, University of Science and Technology of China, Hefei, Anhui,230000, China;

^3^Department of Cardiology,Maanshan People's Hospital,Maanshan Hospital Affiliated to Wannan Medical College,Maanshan, Anhui 243000, China;

^4^Department of Geriatrics Center,Tongling People's Hospital, Tongling, Anhui,244000,China;

^5^Department of Cardiology, The Second People's Hospital of Hefei, Hefei Hospital Affiliated to Anhui Medical University, Hefei, Anhui 230000, China;

***Corresponding Author:**

Zheng Wang,Department of Cardiology, The Second People's Hospital of Hefei, Hefei Hospital Affiliated to Anhui Medical University, Hefei, Anhui 230000, China;E-mail:wang20241007@163.com

^+^:These authors contributed equally and are regarded as co-first authors.

**Supplementary Table 1. Definition of CKM**

| CKM stages | Definition |
| --- | --- |
| CKM stage 0 | Participants with a normal Body Mass Index (BMI) (<23 kg/m² for those of Asian ethnicity and <25 kg/m² for individuals from other racial and ethnic backgrounds), and a normal waist circumference (<80 cm for Asian women and <90 cm for Asian men, or <88 cm for women and <102 cm for men in all other racial and ethnic groups) who did not fulfill the criteria for the other stages. |
| CKM stage 1 | Elevated Body Mass Index (BMI) (≥23 kg/m² for individuals of Asian descent and >25 kg/m² for all other racial and ethnic groups), increased waist circumference (≥80 cm for Asian women and ≥90 cm for Asian men, or ≥88 cm for women and ≥102 cm for men in other racial and ethnic categories), or prediabetes. Prediabetes is defined as a glycated hemoglobin (HbA1c) level of 5.7% to <6.5% or a fasting blood glucose level between 100 mg/dL and <126 mg/dL. |
| CKM stage 2 | Metabolic risk factors or moderate-to-high-risk chronic kidney disease (CKD) as defined by the Kidney Disease: Improving Global Outcomes (KDIGO) criteria, in accordance with AHA recommendations. The qualifying metabolic risk factors encompassed: elevated fasting serum triglycerides (≥135 mg/dL) ; hypertension; diabetes; metabolic syndrome, characterized by the presence of at least three of the following:  increased waist circumference; reduced high-density lipoprotein (HDL) cholesterol levels (<40 mg/dL for men, <50 mg/dL for women); fasting serum triglycerides ≥150 mg/dL; elevated blood pressure (systolic ≥130 mmHg, diastolic ≥80 mmHg, and/or use of antihypertensive medication); prediabetes. |
| CKM stage 3 | Presence of very-high-risk KDIGO CKD stages or a high-estimated 10-year cardiovascular disease (CVD) risk. The 10-year CVD risk was assessed using the AHA PREVENT equations for predicting cardiovascular events. A 10-year CVD risk of 20% or greater was classified as high risk. |
| CKM stage 4 | Reported history of established cardiovascular conditions, including coronary heart disease, angina, myocardial infarction, heart failure, and cerebrovascular accident. |

**Supplementary Table 2. Items in the 49-item Frailty Index and corresponding detailed scoring criteria.**

| **Defects** | **Scoring** |
| --- | --- |
| **Cognition** | |
| 1. Experience confusion/memory problems | Yes = 1  No = 0 |
| **Dependence** | |
| 2. Managing money | Difficulty = 1  No Difficulty = 0 |
| 3. Stooping, crouching, kneeling |  |
| 4. Lifting or carrying |  |
| 5. House chore |  |
| 6. Preparing meals |  |
| 7. Standing up from armless chair |  |
| 8. Getting in and out of bed difficulty |  |
| 9. Using fork, knife, drinking from cup |  |
| 10. Dressing yourself |  |
| 11. Standing for long periods difficulty |  |
| 12. Grasp/holding small objects |  |
| 13. Attending social events |  |
| 14. Push or pull large objects |  |
| 15. Walking for a quarter mile difficulty |  |
| 16. Walking up 10 steps difficulty |  |
| **Depressive Symptoms** | |
| 17. Have little interest in doing things | Nearly every day = 1  More than half the days = 0.66  Several days = 0.33  Not at all = 0 |
| 18. Feeling down, depressed, or hopeless |  |
| 19. Trouble sleeping or sleeping too much |  |
| 20. Feeling tired or having little energy |  |
| 21. Poor appetite or overeating |  |
| 22. Feeling bad about yourself |  |
| 23. Trouble concentrating on things |  |
| **Comorbidities** | |
| 24. Arthritis | Yes = 1  Suspect = 0.5  No = 0 |
| 25. Thyroid problems |  |
| 26. Chronic bronchitis |  |
| 27. Cancer |  |
| 28. Congestive heart failure |  |
| 29. Coronary heart disease |  |
| 30. Angina |  |
| 31. Heart attack |  |
| 32. Stroke |  |
| 33. Blood pressure |  |
| 34. Diabetes |  |
| 35. Weak/failing kidneys |  |
| 36. Urinary Leakage |  |
| **Hospital Utilization and Access to Care** | |
| 37. Self-rated health | Fair or poor = 1  Excellent, very good, or good = 0 |
| 38. Health now compared with 1 year ago | Worse = 1  About the same, Better = 0 |
| 39. Overnight hospital patient in past year | Yes = 1  No = 0 |
| 40. Frequency of healthcare use during the past year | None = 0, 1 to 5 = 0.5, More than 5 = 1 |
| 41. Number of prescribed medications | None = 0, 1 to 4 = 0.5, ≥5 = 1 |
| **Physical Performance and Anthropometry** | |
| 42. Body mass index | <18.5 or ≥30 = 1  25 to 30 = 0.5  18.5 to 25 = 0 |
| 43. Handgrip strength | Male:  For BMI ≤ 24, GS ≤ 29 = 1;  For BMI 24 to 28, GS ≤ 30 = 1;  For BMI >28, GS ≤ 32 = 1.  Female:  For BMI ≤23, GS ≤17 = 1;  For BMI 23 to 26, GS ≤17.3 = 1;  For BMI 26 to 29, GS ≤ 18 = 1;  For BMI>29, GS ≤ 21 = 1. |
| **Laboratory Values** | |
| 44. Glycohemoglobin (%) | 0% to 5.7% = 0, >5.7% = 1 |
| 45. Red blood cell count **(million cells/ml)** | Male: 4.7 to 6.1 = 0, Other = 1  Female: 4.2 to 5.4 = 0, Other = 1 |
| 46. Hemoglobin (g/dL) | Male: 13.5 to 18 = 0, Other = 1  Female: 12 to 16 = 0, Other = 1 |
| 47. Red cell distribution width (%) | 11.6 to 14.6 = 0, Other = 1 |
| 48. Lymphocyte percent (%) | 20 to 40 = 0, Other = 1 |
| 49. Segmented neutrophils percent (%) | 40 to 80 = 0, Other = 1 |

BMI, Body mass index; GS, grip strength.

**Supplementary Table 3.Baseline characteristics according to HGI quartiles**

| **Variables** | **Β-Value** | **VIF** |
| --- | --- | --- |
| **Age** | 0.006 | 1.862 |
| **Lymphocyte** | 0.024 | 1.484 |
| **Monocyte** | 0.034 | 1.566 |
| **Neutrophils** | 0.050 | 1.264 |
| **PIR** | -0.157 | 1.049 |
| **RBC** | -0.069 | 2.243 |
| **Hemoglobin** | -0.114 | 2.444 |
| **Platelet** | 0.021 | 1.267 |
| **FPG** | 0.038 | 3.371 |
| **HbA1c** | 0.086 | 3.456 |
| **BMI** | 0.124 | 1.311 |
| **ALT** | -0.013 | 3.052 |
| **AST** | 0.059 | 2.899 |
| **Albumin** | -0.066 | 1.285 |
| **Creatinine** | 0.030 | 1.776 |
| **UA** | -0.019 | 1.376 |
| **BUN** | 0.064 | 1.737 |
| **TG** | 0.070 | 1.384 |
| **TC** | 0.049 | 2.945 |
| **HDL** | 0.020 | 1.453 |
| **LDL** | -0.064 | 1.084 |
| **eGFR** | -0.052 | 2.755 |

**PIR:Poverty income ratio,BMI:Body mass index,FPG:Fasting plasma glucose,HbA1c:Glycosylated hemoglobin,UA:Uric acid,BUN:Blood urea nitrogen,TG:Triglyceride,TC:Total cholesterol,HDL:High density lipoprotein,LDL:Low density lipoprotein,eGFR:Estimated glomerular filtration rate,RBC:Red blood cell,ALT:Alanine aminotransferase,AST:Aspartate aminotransferase**

**Supplementary Table 4.Optimal hyper-parameter settings for five models based on 10-fold cross-validation and grid search strategy**

| **Methods** | **Optimal hyper-parameters** |
| --- | --- |
| **RF** | 'mtry':3,'trees':414,'min_n':48 |
| **XgBoost** | 'mtry':6,'min_n':14,'tree_depth':2,'learn_rate':0.0196,'loss_reduction':00147,'sample_size':0.918 |
| **LR** | 'C': 10, 'solver': 'liblinear' |
| **LightGBM** | 'mtry':3,'trees':257,'min_n':10,'tree_depth':3,'learn_rate':0.0924,'loss_reduction':0.149 |
| **DT** | 'cost_complexity':0.000190,'tree_depth':7,'min_n':7 |

**Supplementary Table 5.Baseline Characteristics Comparison: Included vs. Excluded Participants Due to Missing Data Characteristic Included Excluded Difference P-value**

| **Characteristic** | **Included(n=6749)** | **Excluded(n=2293)** | **Difference(95%CI)** | **P-value** |
| --- | --- | --- | --- | --- |
| **Demographics:** | | | | |
| **Age, mean (SE)** | 56.55(0.20) | 59.82(0.41) | 3.27(2.38-4.16) | <0.001 |
| **Age ≥65 years, %** | 31.7 | 42.3 | 10.6(8.2-13.0) | <0.001 |
| **Sex, %** |  |  |  | 0.043 |
| Male | 46.87 | 44.12 | -2.75(-5.4-0.1) |  |
| Female | 53.13 | 55.88 | 2.75(-0.1-5.4) |  |
| **Race/Ethnicity, %** |  |  |  | <0.001 |
| Non-Hispanic White | 74.16 | 68.45 | -5.71(18.8-2.6) |  |
| Non-Hispanic Black | 8.68 | 12.84 | 4.16(2.7-5.6) |  |
| Mexican American | 6.28 | 8.92 | 2.64(4-3.9) |  |
| Other | 10.89 | 9.79 | -1.10(-2.8-0.6) |  |
| **Socioeconomic Factors:** | | | | |
| **PIR, mean (SE)** | 3.27(0.05) | 2.84(0.09) | -0.43(-0.62 to -0.24) | <0.001 |
| **Education,%** |  |  |  | <0.001 |
| Less than High School | 14.73 | 28.67 | 13.94(11.5-16.4) |  |
| High School/Equivalent | 22.49 | 26.81 | 4.32(1.8-6.8) |  |
| Some College or Above | 62.77 | 44.52 | -18.25(-21.2 to -15.3) |  |
| **Clinical Characteristics:** | | | | |
| **BMI, mean (SE)** | 29.24(0.12) | 29.87(0.24) | 0.63(0.14-1.12) | 0.012 |
| **Hypertension, %** | 47.06 | 52.12 | 5.06(2.3-7.8) | <0.001 |
| **Diabetes, %** | 19.99 | 27.38 | 7.39(5.2-9.6) | <0.001 |
| **Medication Use:** | | | | |
| **Antihypertensive, %** | 36.35 | 41.27 | 4.92(2.1-7.7) | 0.001 |
| **Antidiabetic, %** | 11.35 | 17.86 | 6.51(4.7-8.3) | <0.001 |
| **Lipid-lowering, %** | 24.00 | 28.94 | 4.94(2.4-7.5) | <0.001 |
| **Laboratory Data Availability:** | | | | |
| **Complete lab profile, %** | 88.45 | 54.32 | -34.13(-36.8 to -31.4) | <0.001 |
| **eGFR <60 mL/min/1.73m², %** | 12.34 | 18.73 | 6.38(4.5-8.3) | <0.001 |
| **Missing Data Patterns:** | | | | |
| **Frailty index items, % missing** | 4.23 | 47.81 | 43.58(41.2-46.0) | <0.001 |
| **Laboratory variables, % missing** | 8.67 | 62.34 | 53.67(51.1-56.2) | <0.001 |
| **Functional assessments, % missing** | 6.89 | 39.27 | 32.38(29.9-34.9) | <0.001 |
| **CKM Stage Distribution, %** |  |  |  | <0.001 |
| Stage 0 | 28.45 | 22.16 | -6.29(-8.9 to -3.7) |  |
| Stage 1 | 31.67 | 29.34 | -2.33(-5.1-0.4) |  |
| Stage 2 | 25.78 | 31.28 | 5.50(2.8-8.2) |  |
| Stage 3 | 14.10 | 17.22 | 3.12(1.1-5.1) |  |

**Supplementary Table 6.Model Performance Comparison Between Different Frailty Index Thresholds**

| **Metric** | **Threshold ≥0.21**  **(n=1,427;21.14%)** | **Threshold ≥0.25**  **(n=892;13.22%)** | **Difference** | **95%CI** |
| --- | --- | --- | --- | --- |
| **Random Forest Model:** | | | | |
| **AUC** | 0.90 | 0.88 | -0.02 | (-0.05,0.01) |
| **Accuracy** | 0.81 | 0.85 | 0.04 | (0.01,0.07) |
| **Precision** | 0.54 | 0.58 | 0.04 | (-0.02,0.10) |
| **Recall** | 0.84 | 0.79 | -0.05 | (-0.11,0.01) |
| **F1 Score** | 0.66 | 0.67 | 0.01 | (-0.04,0.06) |
| **Feature Importance (SHAP Values):** | | | | |
| **PIR** | 0.152 | 0.146 | -0.006 | (-0.015,0.003) |
| **Antihypertensive use** | 0.134 | 0.128 | -0.006 | (-0.018,0.006) |
| **Hemoglobin** | 0.127 | 0.121 | -0.006 | (-0.016,0.004) |
| **Red blood cell count** | 0.119 | 0.115 | -0.004 | (-0.013,0.005) |
| **Albumin** | 0.113 | 0.109 | -0.004 | (-0.012,0.004) |
| **Diabetes** | 0.107 | 0.103 | -0.004 | (-0.011,0.003) |
| **Correlation coefficient of feature rankings: 0.94 (p<0.001)** | | | | |


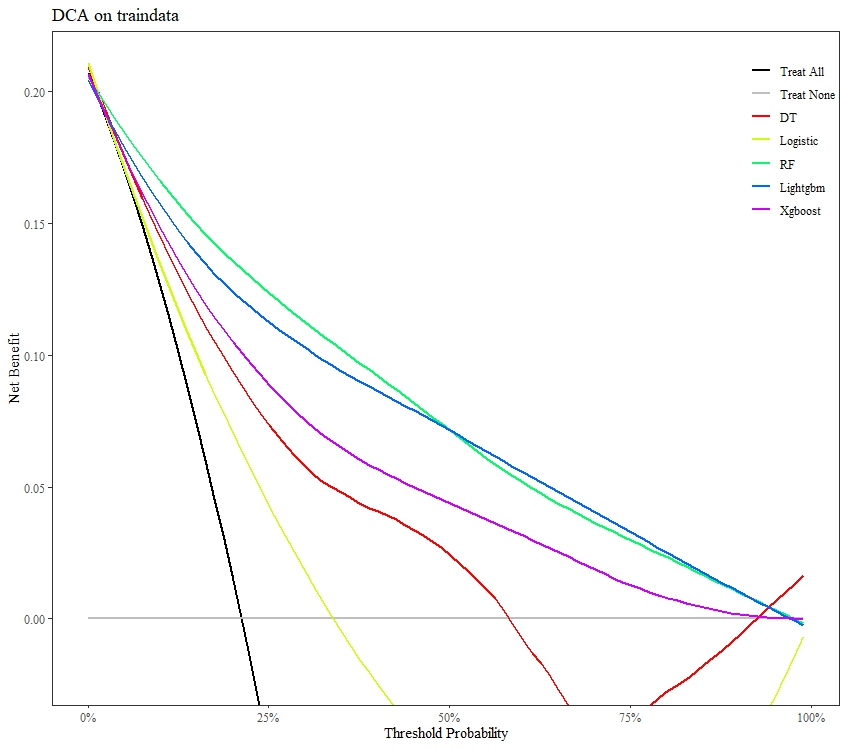

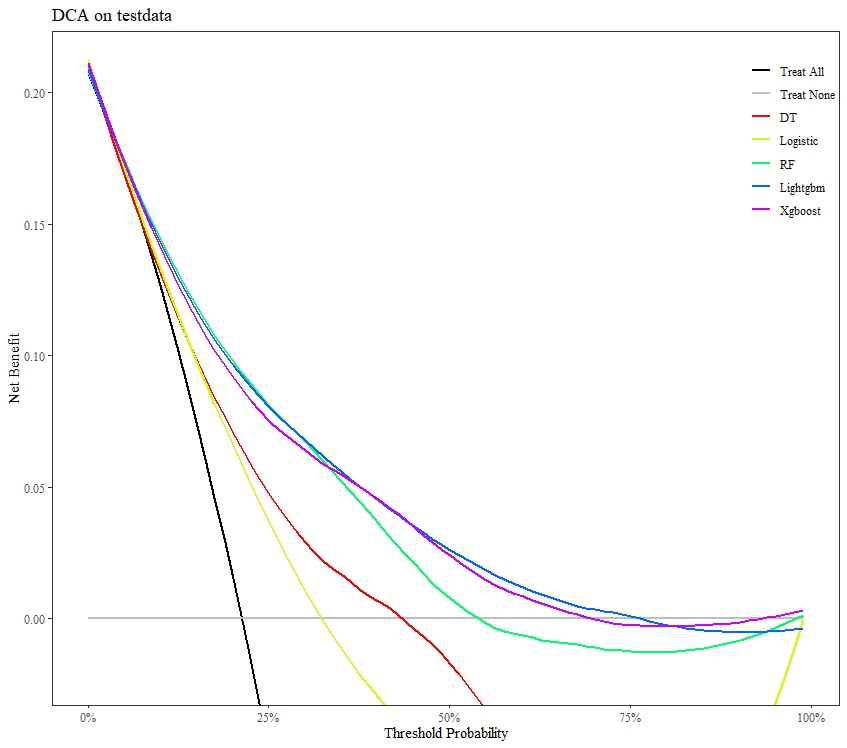


**(A) (B)**

**Supplementary Figure 1.Decision Curve Analysis of Machine Learning Model;(A)Training set(B)Validation Set;DT:Decision Tree,XGBoost:Extreme Gradient Boosting,LR:Logistic Regression,RF:Random forest**


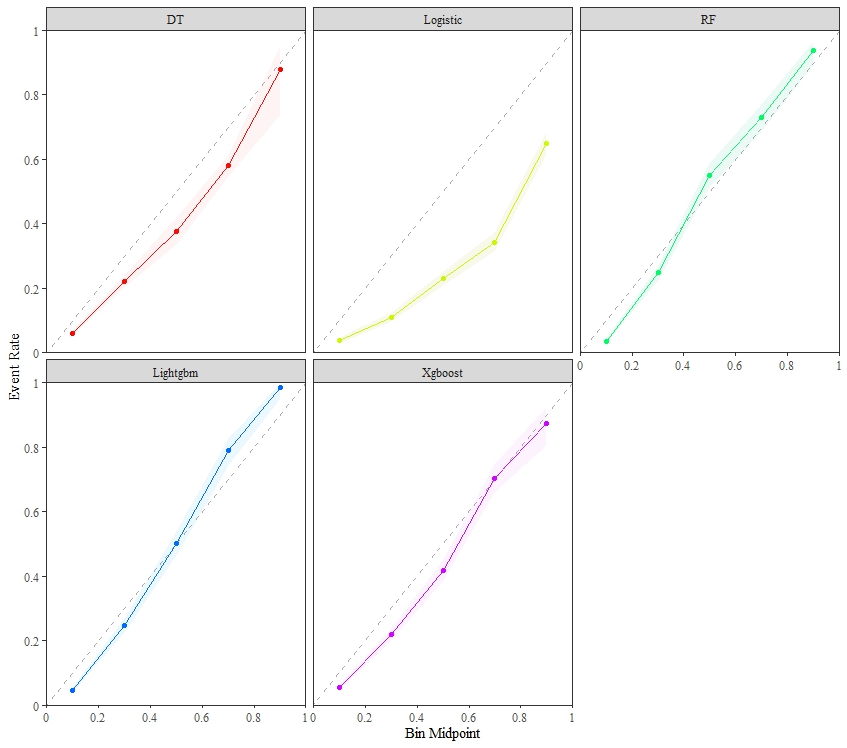

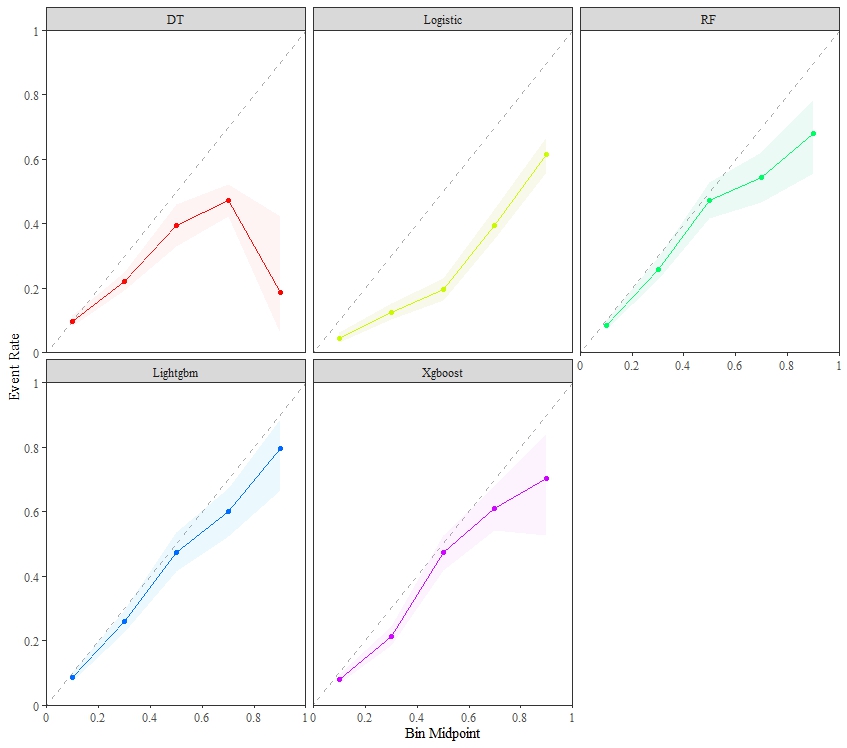


**(A) (B)**

**Supplementary Figure 2.Calibration Curve of Machine Learning Model;(A)Training set(B)Validation Set;DT:Decision Tree,XGBoost:Extreme Gradient Boosting,LR:Logistic Regression,RF:Random forest**
